# Mass spectrometric studies on effects of counter ions of TMPyP4 on binding to human telomeric DNA and RNA G-quadruplexes

**DOI:** 10.1007/s00216-014-7943-0

**Published:** 2014-06-18

**Authors:** Li-Ping Bai, Jie Liu, Li Han, Hing-Man Ho, Renxiao Wang, Zhi-Hong Jiang

**Affiliations:** 1 State Key Laboratory of Quality Research in Chinese Medicine, and Macau Institute for Applied Research in Medicine and Health, Macau University of Science and Technology, Avenida Wai Long, Taipa, Macau; 2 State Key Laboratory of Bioorganic Chemistry, Shanghai Institute of Organic Chemistry, Chinese Academy of Sciences, 345 Lingling Road, Shanghai, 200032 China; 3 School of Chinese Medicine, Hong Kong Baptist University, Kowloon Tong, Kowloon, Hong Kong

**Keywords:** G-quadruplex, Telomere, TMPyP4, Mass spectrometry

## Abstract

A comparative study on human telomeric DNA G-quadruplex binding of meso-5,10,15,20-tetrakis(*N*-methyl-4-pyridyl)porphyrin (TMPyP4) between its two salt forms, i.e., tetratosylate and tetrachloride, was conducted by using ESI-TOF-MS, UV-melting measurement, and molecular modeling methods. Besides cation TMPyP4, the tosyl anion was found to bind to human telomeric DNA G-quadruplex with multiple binding stoichiometries from 1:1 to 3:1 observed in ESI-TOF-MS spectra, indicating that the stabilization activity of TMPyP4 tetratosylate on G-quadruplex is derived from a synergetic effect of both TMPyP4 cation and tosyl anion. A molecular modeling study suggests that a tosyl anion fills up the vacant space between TMPyP4 cation and DNA G-quadruplex and thus stabilizes the complex by 3.8 kcal/mol. Therefore, it is estimated that TMPyP4 tetratosylate’s activity might not reflect the real effect of TMPyP4 cation in some bioassays related to G-quadruplex stabilization. This was verified by the results of less binding affinity of TMPyP4 tetrachloride with DNA G-quadruplex obtained from ESI-TOF-MS measurement, and of 2.27 °C less thermal stabilization of TMPyP4 tetrachloride for DNA G-quadruplex, compared to its tetratosylate under the same conditions. Our study demonstrated the influence of counter ions of TMPyP4 on G-quadruplex binding, which sheds light on the proper usage of TMPyP4 salt in the chemical and biological research associated with G-quadruplex binding. Subsequently, the binding of TMPyP4 tetrachloride to human telomeric RNA G-quadruplexes was studied with ESI-TOF-MS technique. The binding constants of TMPyP4 with human telomeric G-quadruplexes indicated that TMPyP4 binds to human telomeric RNA G-quadruplex one order of magnitude stronger than DNA counterpart. This is a comprehensive mass spectrometric report on binding study of TMPyP4 with human telomeric DNA/RNA G-quadruplexes.

**Electronic supplementary material:**

The online version of this article (doi:10.1007/s00216-014-7943-0) contains supplementary material, which is available to authorized users.

**Figure:**
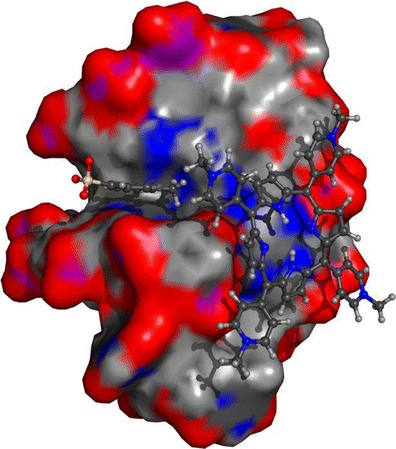
ᅟ

## Introduction

As a wide spectrum of G-quadruplex binder, meso-5,10,15,20-tetrakis(*N*-methyl-4-pyridyl)porphyrin (TMPyP4, see Electronic Supplementary Material Fig. S1) has been extensively studied in the aspects of G-quadruplex binding [1–6] and inducing activities [7, 8], telomerase inhibition [1, 3, 9–12], and anticancer effects [10–14]. The antitumor activity of TMPyP4 tetratosylate (see Electronic Supplementary Material Fig. S1) has been examined in K562 leukemic cells, retinoblastoma cell lines, and osteosarcoma cell lines. Regarding the mechanism of its anticancer activity, it has been show that TMPyP4 tetratosylate significantly inhibited telomerase activity in telomerase positive HOS and Saos-2 cells, induced telomere shortening, and inhibited the cell growth in HOS and Saos-2 cells with over 17 % apoptosis rates. These indicated that antitumor effect of TMPyP4 may be related to telomere dysfunction through G-quadruplex stabilization [14].

It was noted that TMPyP4 has been mostly used in its salt form of tetratosylate from commercial supplier as a G-quadruplex ligand in many literature reports [2, 12–21]. The counter ion tosyl may contribute to G-quadruplex stabilization effect of TMPyP4 due to a consideration that its planar molecule may also interact with G-quadruplex. To attest our prediction, G-quadruplex binding study was conducted between two salt forms of TMPyP4, i.e., tetratosylate and tetrachloride, by using ESI-QTOF-MS, UV-melting measurement, and molecular modeling studies. The G-quadruplex binding affinities of these two salt forms of TMPyP4 with human telomeric DNA were compared. In addition, G-quadruplex binding activity of TMPyP4 tetrachloride was also quantitatively compared between two types of human telomeric G-quadruplexes, i.e., DNA and its RNA anologue, for a purpose of exploring its binding specificity with nucleic acid G-quadruplexes.

## Experimental procedures

### Sample preparation

Amberlite® IRA-400 (Cl) ion exchange resin was purchased from Aldrich Chemical Company. TMPyP4 tetratosylate from Sigma-Aldrich (USA) was converted to the tetrachloride salt as follows. TMPyP4 tetratosylate (5 mg) dissolved in Milli-Q water was loaded to an Amberlite® IRA-400 (Cl) ion exchange resin column (2.5 cm × 15 cm) and eluted with Milli-Q water to afford TMPyP4 tetrachloride (2.32 mg) with a yield of 75.1 %. TMPyP4 tetrachloride was identified by ^1^H-NMR technique (see Electronic Supplementary Material Fig. S2).

### ESI-TOF-MS spectrometry

All ESI-TOF-MS experiments were performed on a Bruker MicrOTOF-Q mass spectrometer. The experimental conditions were optimized to avoid denaturation of the G-quadruplex species. The capillary voltage was +3,500 V and the dry gas N_2_ flow was 4.0 L/min at 100 °C. The analyzer was operated at a background pressure of 3 × 10^−7^ mbar. The rate of sample infusion into the mass spectrometer was 3 μL/min. Data were analyzed with the instrument software Bruker Daltonics DataAnalysis. All nucleic acid oligomers (desalted grade) d[(TTAGGG)_4_TTA] (*M* = 8496.6 Da) and r[(UUAGGG)_4_UUA] (*M* = 8789.3 Da) were purchased from Invitrogen (Japan) and used without further purification. The stock solutions of all nucleic acid oligomers were prepared in Milli-Q water at the concentration of 1 mM, and further diluted by 1 M NH_4_OAc buffer (pH 7.6) to the desired concentration. All stock solutions of TMPyP4 were prepared in Milli-Q water with a concentration of 400 μM. The samples of an equal molar mixture of DNA/RNA oligomer and TMPyP4 were prepared in 100 mM NH_4_OAc (pH 7.6), and then were kept in room temperature for 30 min. A final strand concentration of 5 μM (or 10 μM) DNA/RNA oligomer and 5 μM (or 10 μM) TMPyP4 in 50 mM NH_4_OAc (pH 7.6) containing 50 % methanol were flow injected into mass spectrometer for the measurement of binding affinity [22, 23].

### ESI-QTOF-MS spectrometry

All QTOF-MS/MS experiments were carried out on a Bruker maXis impact mass spectrometer in the negative mode. The source parameters were optimized as follows: capillary voltage of +3,500 V, nebulizer of 1.5 bar, dry gas flow of 4.0 L/min at 160 °C, and end plate offset voltage of 500 V. The tune parameters were set as follows: 400 Vpp of funnel 1 RF, funnel 2 RF, and hexapole RF for transfer parameters; 5 eV of ion energy and 350 *m*/*z* of low mass for quadrupole parameters; 10 eV of collision energy, 1,500 Vpp of collision RF, 180 μs of transfer time, and 16 μs of prepulse storage time for collision cell parameters; 0.1 Hz of spectra rate, mass range from 300 to 3,000 *m*/*z*, and 20 Da of width for MS/MS parameters. Each QTOF-MS/MS spectrum was an average of at least 30 scans.

### UV-melting measurement

Thermal denaturation was carried out in 25 mM Tris–HCl buffer (pH 7.4) containing 100 mM KCl on a UV-2700 spectrometer (Shimadzu, Japan) equipped with a Shimadzu TMSPC-8 temperature controller. The absorbance was determined at 295 nm, while the temperature was programmed to increase from 20 to 95 °C with a heating rate of 1 °C/min. The DNA concentration was 5 μM for the Δ*T*_m_ measurements of dAGGG(TTAGGG)_3_ in the absence or presence of either TMPyP4 tetratosylate or TMPyP4 tetrachloride in a 2:1 ligand/DNA molar ratio. The melting temperature (*T*_m_) was the average value from three independent measurements [24].

### Molecular modeling

**Fig. 1:**
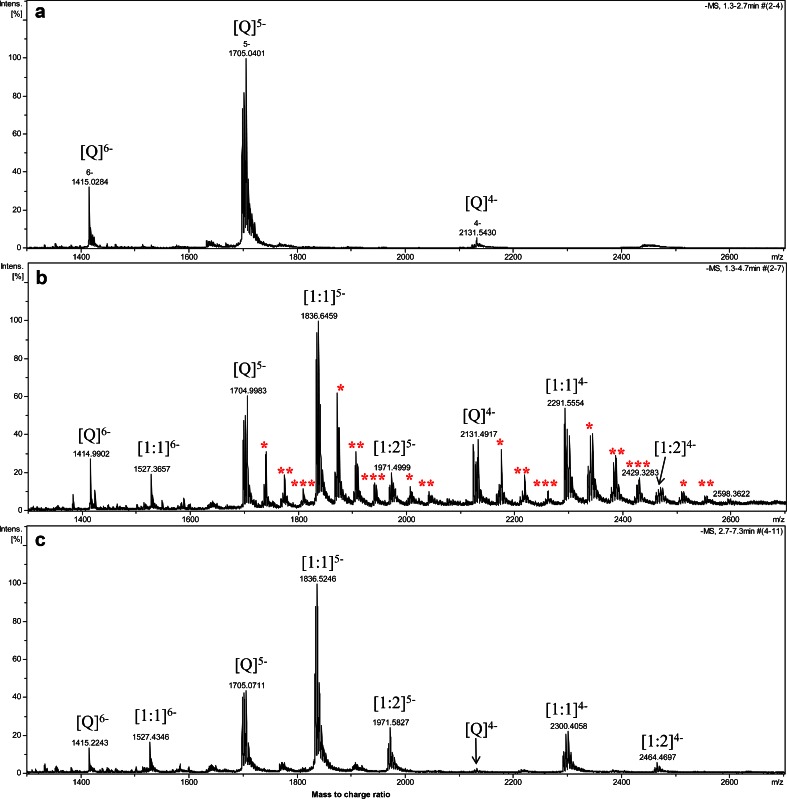
Negative ESI-TOF-MS spectra of human telomeric DNA sequence d[(TTAGGG)_4_TTA] (Q) alone (**a**), with equimolar mixture of TMPyP4 tetratosylate (**b**), and TMPyP4 tetrachloride (**c**). Spectra were recorded with 1:1 DNA-to-drug molar ratio (*C* = 10 μM) in 50 mM ammonium acetate buffer (pH 7.6) containing 50 % methanol. [1:1]^5−^ means the complex of Q and TMPyP4 cation of 1:1 molar ratio in −5 charge state; [1:2]^5−^ means the complex of Q and TMPyP4 cation of 1:2 molar ratio in −5 charge state, and similarly as to the others. The symbol *, **, and *** represent the number of tosyl adducts 1, 2, and 3, respectively

**Fig. 2:**
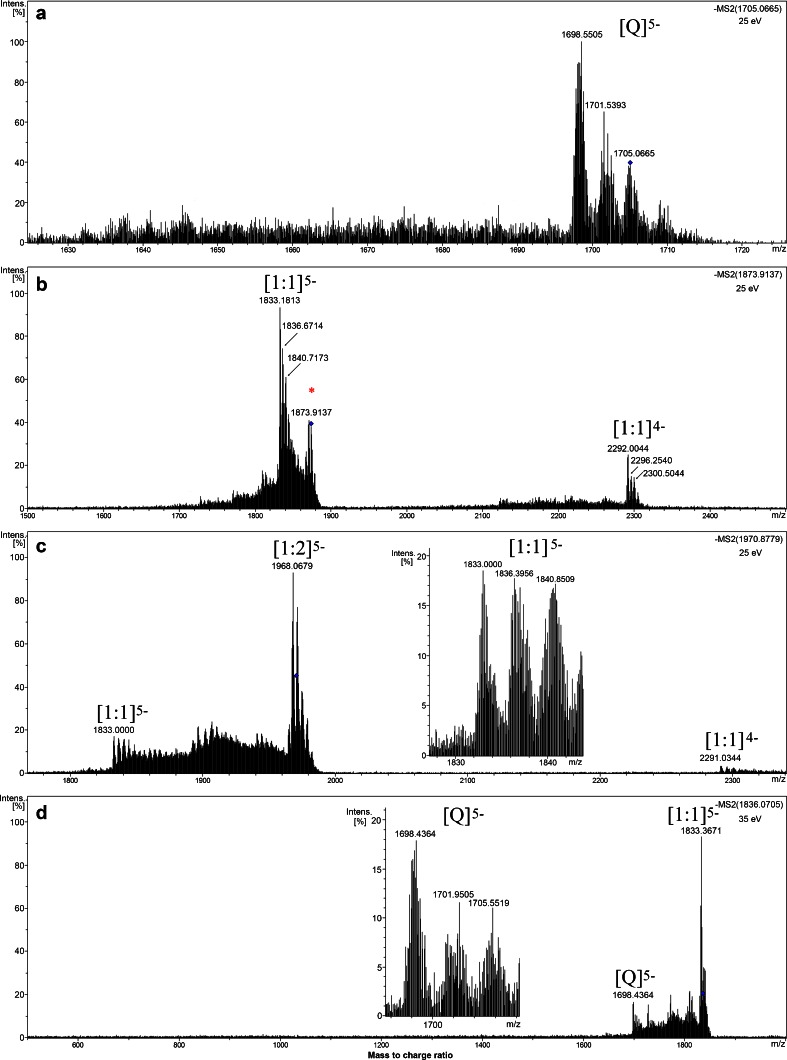
Negative ESI-QTOF-MS/MS spectra of human telomeric DNA G-quadruplex (Q) precursor ions at *m*/*z* 1,705.0665 (**a**), 1:1:1 complex of G-quadruplex:TMPyP4:tosyl anion at *m*/*z* 1873.9137 (**b**), 1:2 complex of G-quadruplex:TMPyP4 at *m*/*z* 1970.8779 (**c**), and 1:1 complex of G-quadruplex :TMPyP4 at *m*/*z* 1836.0705 (**d**). Spectra were recorded with 1:1 molar ratio (*C* = 50 μM) of d[(TTAGGG)_4_TTA] and TMPyP4 tetratosylate in 10 mM ammonium acetate buffer (pH 7.6) containing 50 % methanol. The collision energy was 25 eV for **a**–**c**, and 35 eV for **d**. [1:1]^5−^ means the complex of Q and TMPyP4 cation of 1:1 molar ratio in the −5 charge state, and similarly as to the others. *Asterisk* represents the tosyl anion adduct. The inserts in **c** and **d** are the enlargement of [1:1]^5−^ and [Q]^5−^, respectively

Binding mode of TMPyP4 cation and tosyl anion to the human telomeric DNA G-quadruplex was derived through molecular modeling. A three-dimensional structure of human telomeric DNA G-quadruplex in the Protein Data Bank (PDB entry 2JPZ) [25] was used as the template to construct the structural model of the DNA G-quadruplex used in our experimental study. Molecular docking of TMPyP4 cation to G-quadruplex was performed by using the GLIDE module in the Schrödinger software (version 9.3.5, released by the Schrödinger Inc.). The binding pocket was defined to consist of residues A3, A15, G16, and A21, which locates roughly at the top of the 5′-terminal of the G-quadruplex. The size of the binding pocket was set to 20 Å. The GlideScore scoring function in the SP mode was applied to evaluate possible binding poses of TMPyP4 cation. Only the binding pose with the highest binding score was selected for further consideration. On account of G-quadruplex is negatively charged and TMPyP4 positively charged, the tosyl anion was manually docked upon the position of TMPyP4 cation (Figs. 1 and 2).

The two models of TMPyP4 cation in complex with G-quadruplex (with or without the tosyl anion) were then refined by molecular dynamics (MD) simulations. Both MD jobs were conducted by using the AMBER program (version 12, released by UCSF). Small-molecule ligands (i.e., TMPyP4 and tosyl) were prepared by the Antechamber module in AMBER. Atomic partial charges on small-molecule ligands and G-quadruplexes were both computed with the Gaussian software (version 03, released by the Gaussian Inc) at the HF/6-31G* level and then assigned by the RESP method. All ionizable bases on the G-quadruplexes were set at their default deprotonation states at a neutral pH. A number of counter ions were added to neutralize the whole molecular system. The whole complex was then soaked at the center of a TIP3P water box with a margin of 10 Å along each dimension. After minimization of the whole system, a MD simulation of 20 ns long was conducted for each complex at a constant pressure of 1 atm and a constant temperature of 300 K. The time interval in each MD job was set to 2 fs. The SHAKE method was applied to restrict all covalent bonds connecting hydrogen atoms on small-molecule ligands as well as G-quadruplex. In addition, a harmonic restraint of 50 kcal/mol Å^2^ was applied to all heavy atoms on G-quadruplex. Potential energies were calculated by using the AMBER FF10 force field with a nonbonded distance cutoff of 10 Å. The Particle Mesh Ewald (PME) method was adopted to compute long-range electrostatic interactions. The last snapshot on each resulting MD trajectory was retrieved as the final complex model shown in Fig. 4.

Based on the obtained MD trajectories of two TMPyP4/G-quadruplex complexes, the MM-GB/SA method in AMBER was employed to compute the binding free energy between TMPyP4 cation and G-quadruplex with and without the presence of the tosyl anion. A total of 1,000 snapshots were retrieved from the 10–20-ns segment on each MD trajectory with an interval of 10 ps. Binding free energies were computed on this configuration ensemble, and the average value was recorded as the final result.

## Results

### Binding of two salt forms of TMPyP4 to human telomeric DNA G-quadruplex

ESI-TOF-MS is a powerful tool for directly observing the binding affinity of small molecules with biomolecules such as DNA and RNA. This effective and rapid analytical technique has been playing an active role in the investigations of noncovalent complexes involving biopolymers [26]. Moreover, binding constants for ligands with oligodeoxynucleotides including G-quadruplex has been reliably measured by ESI-MS [27, 28].

**Fig. 3:**
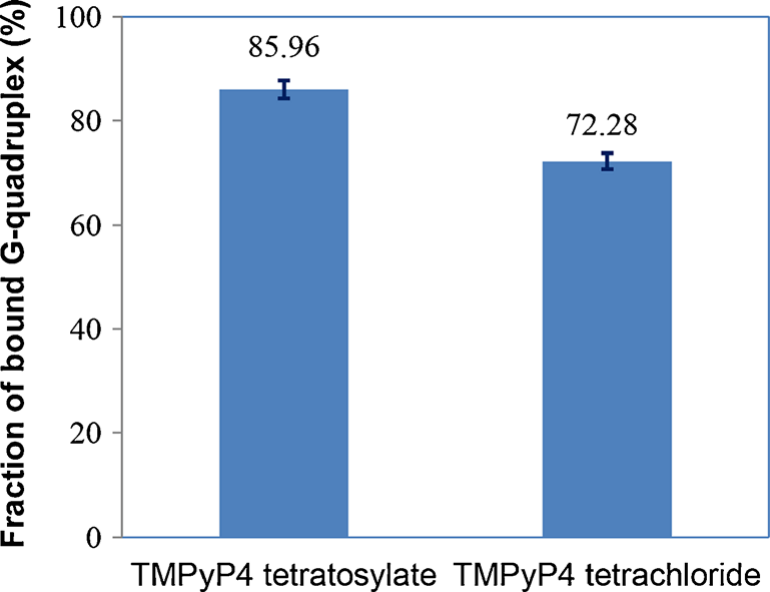
The fraction of bound G-quadruplex of TMPyP4 tetratosylate and TMPyP4 tetrachloride with human telomeric DNA G-quadruplex (*n* = 3)

**Fig. 4:**
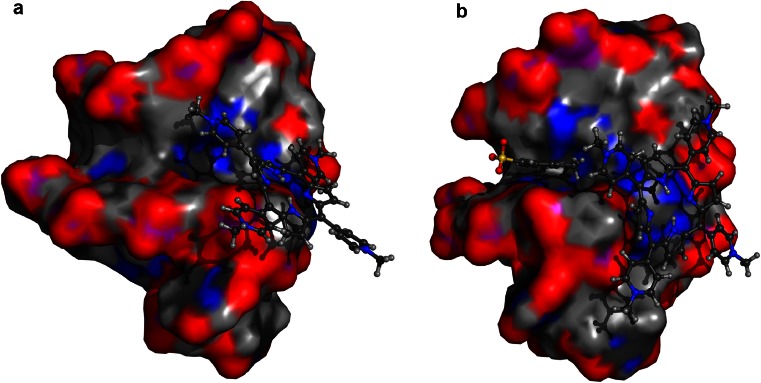
Structural models of TMPyP4 binding with human telomeric G-quadruplex in the absence (**a**) and presence (**b**) of one tosyl ion, which were derived through molecular dynamics simulations. Computed binding energies of TMPyP4 with telomeric G-quadruplex are −27.6 (**a**) and −31.4 kcal/mol (**b**), respectively. TMPyP4 and tosyl ion are shown in the ball-and-stick model, whereas telomeric G-quadruplex is shown in the molecular surface model

In order to directly observe the influence of tosyl group on the G-quadruplex binding of TMPyP4, ESI-TOF-MS was utilized to examine the binding of TMPyP4 tetratosylate with human telomeric DNA G-quadruplex d[(TTAGGG)_4_TTA]. As illustrated in Fig. 1, the ESI-TOF-MS spectrum of telomeric DNA d[(TTAGGG)_4_TTA] showed that adding of the NH_4_OAc buffer facilitated the detection of quadruplex (Q^5−^ in Fig. 1a) [24] in the predominant −5 charge state ion at *m*/*z* 1,705.0401, which corresponds to two NH_4_^+^ ion adducts of the oligodeoxynucleotide d[(TTAGGG)_4_TTA]. This indicated that G-quadruplex can maintain their structure in gas phase because two ammonium cations were sandwiched between three G-tetrads of the G-quadruplex structure [29–32]. When TMPyP4 tetratosylate was added to above G-quadruplex DNA (Fig. 1b), 1:1 and 1:2 complex peaks of G-quadruplex with TMPyP4 cation were observed in three charge states of −4, −5, and −6. Interestingly, a series of tosyl adducts were simultaneously detected in both TMPyP4-free G-quadruplex and TMPyP4-G-quadruplex complexes with multiple binding stoichiometries from 1:1 to 3:1, indicating that tosyl anion additionally contributed to G-quadruplex stabilization effect of TMPyP4 tetratosylate. QTOF-MS/MS was utilized to further identify the nature of the above complexes (Fig. 2). As shown in Fig. 2a, the precursor ion of free G-quadruplex at 1,705.0665 (with 2 ammoniums adducts) generated 2 daughter ions at 1,701.5393 (with 1 ammonium adduct) and 1,698.5505 (without ammonium ion) under the collision energy of 25 eV. The MS/MS of the 1:1:1 complex of G-quadruplex:TMPyP4:tosyl anion at *m*/*z* 1,873.9137 under the collision energy of 25 eV produced daughter ions of 1:1 G-quadruplex-TMPyP4 complex with 0–2 ammonium adducts in the charge state of −5 (at *m*/*z* of 1,840.7173, 1,836.6714, and 1,833.1813, respectively) and −4 (at *m*/*z* of 2,300.5044, 2,296.2540, and 2,292.0044, respectively), but without any daughter ions of the free G-quadruplex (Fig. 2b). The daughter ions of 1:1 complex of G-quadruplex and TMPyP4 were also produced by the MS/MS of the precursor ion of 1:2 complex of G-quadruplex and TMPyP4 at *m*/*z* 1,970.8779 with the collision energy of 25 eV (Fig. 2c). When a collision energy of at least 35 eV was used to the precursor ion of 1:1 complex of G-quadruplex-TMPyP4 at *m*/*z* 1,836.0705, daughter ions of the free G-quadruplex (at *m*/*z* 1,698.4364, 1,701.9505, and 1,705.5519) can be observed (Fig. 2d), indicating that a higher collision energy is required to break away the TMPyP4 cation from the 1:1 complex of G-quadruplex-TMPyP4. In order to investigate the real binding of TMPyP4 cation with G-quadruplex, the counter ion of tosyl in the commercial agent TMPyP4 tetratosylate was replaced by chloride ion by an Amberlite® IRA-400 (Cl) ion exchange column chromatography. The binding study of TMPyP4 tetrachloride to human telomeric DNA G-quadruplex was subsequently carried out under the same ESI-TOF-MS condition as that for TMPyP4 tetratosylate (Fig. 1c). To compare the G-quadruplex binding affinity of two salt forms of TMPyP4 with human telomeric sequence, the fraction of bound G-quadruplex, i.e., the peak area ratio of all [complex] to total G-quadruplex ([complex] and [free G-quadruplex]) [27, 33, 34], was used to evaluate the relative binding affinities (RBA; Fig. 3). As shown in Fig. 3, TMPyP4 tetrachloride displayed a relatively less binding affinity than that of TMPyP4 tetratosylate, which was regarded to be ascribable to the contribution of tosyl anion’s binding to DNA G-quadruplex. To provide supporting evidence, the UV-melting measurement was utilized to compare the thermal stabilizing effect of two salts of TMPyP4 on the DNA G-quadruplex. As a result (see Electronic Supplementary Material Fig. S3), the melting temperature (*T*_m_) of DNA G-quadruplex was increased 6.45 and 4.18 °C by TMPyP4 tetratosylate and its tetrachloride, respectively, under the same condition. An extra increase of 2.27 °C by TMPyP4 tetratosylate in the Δ*T*_m_ of DNA G-quadruplex further confirmed that the tosyl anion contributes to the stabilizing effect of TMPyP4 tetratosylate on DNA G-quadruplex. Our molecular dynamics simulations showed that a tosyl anion tends to stay inside a narrow groove on the surface of G-quadruplex and beside where TMPyP4 cation binds (Fig. 4). The tosyl anion fills up the vacant space between G-quadruplex and TMPyP4 and thus stabilizes the whole complex. Indeed, computed binding energy of TMPyP4 to G-quadruplex in the presence of the tosyl anion (−31.4 kcal/mol) is 3.8 kcal/mol lower than the result of the same complex when the tosyl anion is absent (−27.6 kcal/mol). Therefore, it indicates that TMPyP4 tetratosylate may not be an appropriate positive control used as a G-quadruplex ligand in bioassays, especially for the quantitative measurements, such as solution measurement by kinetic analysis methods including UV–VIS spectrophotometry [15–17], CD spectroscopy [16, 17, 21], surface plasmon resonance (SPR), and DNA polymerase stop assay [18] etc., as well as the antitumor assay involving G-quadruplex stabilization of TMPyP4 [12–14]. An inorganic salt form of TMPyP4, such as its tetrachloride [1, 3, 5, 6], reflecting the real interaction of TMPyP4 cation with G-quadruplex, should be employed as G-quadruplex binder for quantitative assays.

### Binding of TMPyP4 to human telomeric RNA G-quadruplex

**Fig. 5:**
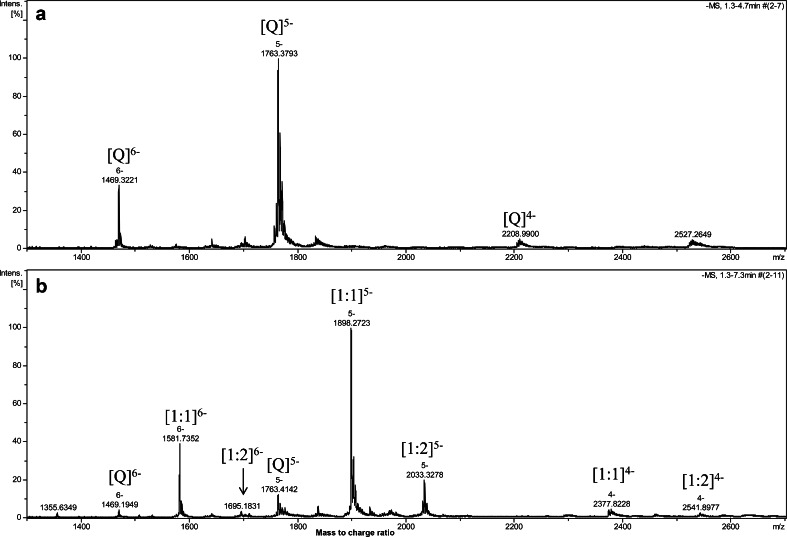
Negative ESI-TOF-MS spectra of human telomeric RNA sequence r[(UUAGGGG)_4_UUA] (Q) alone (**a**) and with equimolar mixture of TMPyP4 tetrachloride (**b**). Spectra were recorded with 1:1 RNA-to-TMPyP4 molar ratio (*C* = 10 μM) in 50 mM ammonium acetate buffer (pH 7.6) containing 50 % methanol. [1:1]^5−^ means the complex of Q and TMPyP4 cation of 1:1 molar ratio in −5 charge state; [1:2]^5−^ means the complex of Q and TMPyP4 cation of 1:2 molar ratio in −5 charge state, and similarly as to the others

Recently, it was reported that human telomeric repeat-containing RNA sequence r(UUAGGG)_4_ also folds into a parallel G-quadruplex in solution which is more stable than its DNA counterparts [35–38]. RNA G-quadruplexes are present in transcripts associated with telomeres and play important roles in key cellular functions, including telomere homeostasis and gene expression [39]. Since TMPyP4 tetrachloride is recommended as a better positive control for G-quadruplex-related bioassay on the basis of our ESI-TOF-MS, UV-melting, and molecular modeling results, the binding of TMPyP4 tetrachloride with human telomeric RNA G-quadruplex r[(UUAGGG)_4_UUA] was therefore studied under the same ESI-TOF-MS condition as that for DNA counterpart for a comparative purpose. As shown in the ESI-TOF-MS spectrum of r[(UUAGGG)_4_UUA] (Fig. 5a), the two predominant ions with *m*/*z* value of 1,469.3221 and 1,763.3793 correspond to two NH_4_^+^ ion adducts of r[(UUAGGG)_4_UUA] in the charge state of −6 and −5, respectively. This suggested the high stability of RNA G-quadruplex in the gas phase. After adding an equal molar of TMPyP4 tetrachloride to RNA G-quadruplex (Fig. 5b), the complex of [1:1] and [1:2] stoichiometries of G-quadruplex with TMPyP4 became predominant peaks, while the free RNA G-quadruplex presents in very minor peaks, showing the extremely strong binding of TMPyP4 with RNA G-quadruplex.

In order to quantitatively compare the binding capacity of TMPyP4 cation between two types of G-quadruplex, i.e., human telomeric RNA G-quadruplex and its DNA counterpart, the equilibrium association constants *K*_1_ and *K*_2_ of TMPyP4 with both human telomeric RNA and DNA G-quadruplexes were calculated according to the literature method [27]. The relative intensities or peak areas of the free and bound G-quadruplex in the mass spectra are assumed to be proportional to the relative abundances of these species in solution. As the initial concentrations are known, the concentrations of all individual species at equilibrium (free G-quadruplex, 1:1 complex, 1:2 complex, and by difference, the free ligand) can be determined from the relative intensities or peak areas of the free G-quadruplex and the complexes. Therefore, the equilibrium association constants *K*_1_ and *K*_2_ were calculated directly from the following Eqs. 1 and 2, respectively [27].

1 $$ {K}_1=\left[1:1\ \mathrm{complex}\right]/\left(\left[\mathrm{free}\ \mathrm{G}-\mathrm{quadruplex}\right]\kern0.5em \left[\mathrm{free}\ \mathrm{drug}\right]\right) $$

2 $$ {K}_2=\left[1:2\kern0.5em \mathrm{complex}\right]/\left(\left[1:1\ \mathrm{complex}\right]\kern0.5em \left[\mathrm{free}\ \mathrm{drug}\right]\right) $$

The binding constants were determined at an equimolar mixture of TMPyP4 tetrachloride and G-quadruplex (10 μM:10 μM and 5 μM:5 μM, respectively). As a result (Table 1), binding affinities (*K*_1_ and *K*_2_) of TMPyP4 with RNA G-quadruplex were at least tenfold higher than those with DNA G-quadruplex. The binding affinities (*K*_1_) 1.87 × 10^6^ M^−1^ and (*K*_2_) 2.83 × 10^5^ M^−1^ of TMPyP4 with human telomeric DNA G-quadruplex calculated by ESI-TOF-MS analysis were almost consistent with those reported (*K*_1_) 2 × 10^6^ M^−1^ and (*K*_2_) 5 × 10^5^ M^−1^ by ITC experiments [40]. This indicated that the binding affinity measured by gas-phase method of ESI-TOF-MS is comparable to that acquired by solution-phase method.

**Table 1:** Equilibrium association constants (*K*) of TMPyP4 tetrachloride with the intramolecular telomeric G-quadruplex obtained by ESI-TOF-MS analysis (*n* = 3)

Ligand	d[(TTAGGG)_4_TTA]		r[(TTAGGG)_4_TTA]	
	*K*_1_ (M^−1^)	*K*_2_ (M^−1^)	*K*_2_ (M^−1^)	*K*_2_ (M^−1^)
TMPyP4 tetrachloride	(1.87 ± 0.19) × 10^6^	(2.83 ± 0.29) × 10^5^	(5.62 ± 0.71) × 10^7^	(2.62 ± 0.27) × 10^6^

## Discussion

The binding of commercial telomerase inhibitor TMPyP4 tetratosylate with human telomeric DNA G-quadruplex was examined by ESI-TOF-MS technique in this study. The counter ion tosyl was found to bind to G-quadruplex DNA in a series of multiple binding stoichiometries from 1:1 to 3:1, which contributes to 13.68 % (85.96–72.28 %) RBA value of TMPyP4 tetratosylate more than that of its tetrachloride. UV-melting study showed that TMPyP4 tetratosylate increased 2.27 °C more than its tetrachloride in the Δ*T*_m_ value of DNA G-quadruplex, further supporting the contribution of tosyl anions to the thermal stability of TMPyP4-bound DNA G-quadruplex. The computed binding energy of the 1:1:1 complex of G-quadruplex:TMPyP4:tosyl anion is favorable by −3.8 kcal/mol as compared to the result of the 1:1 complex of DNA G-quadruplex with TMPyP4. This finding alerts that the commercial TMPyP4 tetratosylate may not be a suitable agent used for TMPyP4’s bioassays related to G-quadruplex stabilization [12–19], and proposed the usage of TMPyP4 tetrachloride as a better one for G-quadruplex research. Our study demonstrated the influence of counter ions of TMPyP4 on G-quadruplex binding, which shed light on the proper usage of TMPyP4 salt in the chemical and biological research associated with G-quadruplex binding. It should be mentioned that it is worthwhile to explore the influences of different counter ions (tosylate and chloride) on biological and pharmacological activity of TMPyP4 in G-quadruplex-related bioassays, such as antitumor and cytotoxic assays.

In addition, the binding of TMPyP4 tetrachloride with both human telomeric DNA and RNA G-quadruplexes was also quantitatively carried out by ESI-TOF-MS analysis. Both 1:1 and 2:1 binding stoichiometries were observed for TMPyP4 binding to two types of G-quadruplexes. The binding constants (*K*_1_) and (*K*_2_) of TMPyP4 tetrachloride with DNA G-quadruplex derived from ESI-TOF-MS analysis were almost consistent with those obtained by solution method of ITC experiments [40], indicating that ESI-TOF-MS analysis is an alternative method to reliably measure G-quadruplex binding constant. The binding constant of *K*_1_ is one order of magnitude stronger than *K*_2_ for TMPyP4 tetrachloride binding to human telomeric DNA and RNA G-quadruplexes. A similar case was reported for TMPyP4 binding to a human *c*-*kit* proto-oncogene DNA G-quadruplex with a strong binding constant (*K*_1_) around 10^7^, and a weak *K*_2_ around 10^6^ [41]. The first binding event (*K*_1_) is typical of an interaction via end stacking [29, 40, 42], while the second binding event (*K*_2_) is probably due to the interaction via groove or external loop binding [41, 42]. The QTOF-MS/MS results supported the above speculation that a higher collision energy of at least 35 eV is required to break away the TMPyP4 cation from the precursor ion of 1:1 complex of G-quadruplex-TMPyP4 (Fig. 2d), while a weaker collision energy of 25 eV is enough to drive out one TMPyP4 cation with the weaker G-quadruplex-binding affinity from the precursor ion of 1:2 complex of G-quadruplex-TMPyP4 (Fig. 2c). In addition, no any daughter ions of the free G-quadruplex were detected in Fig. 2c, indicating that the collision energy of 25 eV is too weak to simultaneously exclude two TMPyP4 cations from the precursor ion of 1:2 complex of G-quadruplex-TMPyP4.

ESI-TOF-MS is a powerful and reliable analytical tool in the study of the noncovalent interactions between TMPyP4 and nucleic acids with inherent advantages of speed, specificity, sensitivity, and stoichiometry [21, 22, 24, 26–28], compared with the conventional spectroscopic methods such as UV spectrophotometry, fluorospectrophotometry and circular dichroism [2, 6, 8, 15–18, 43], etc. Firstly, the rapid measurement of a mass spectrum within 2 min allows the accurate determination and direct observation of binding stoichiometries of TMPyP4 with nucleic acids, which is impossible by the methods of UV spectrophotometry, fluorospectrophotometry, and circular dichroism. Secondly, the high-resolution ESI-MS data show great superiority of precisely sorting out all different species of nucleic acids simultaneously, which provides more comprehensive information on the binding of TMPyP4 cation and tosyl anion with nucleic acids. But only the weighted contribution of all species present in solution is measured in the conventional spectroscopic methods. Thirdly, the relative binding affinities or even equilibrium constants obtained by ESI-TOF-MS usually match closely the ranking order obtained from other solution methods. Finally, QTOF-MS/MS can help to identify the nature of complexes of drug with nucleic acids.

## Conclusion

Based on the results obtained from ESI-TOF-MS, ESI-QTOF-MS/MS, UV-melting measurement, and molecular modeling, it was concluded that the tosyl anion of commercial TMPyP4 tetratosylate contributes to the G-quadruplex stabilizing effect. This finding shed light on the proper usage of TMPyP4 salt in the chemical and biological research associated with G-quadruplex binding. In this study, ESI-TOF-MS technique provided direct evidence that TMPyP4 cation binds to DNA/RNA G-quadruplex with both 1:1 and 2:1 binding stoichiometries. Moreover, the binding capacity of TMPyP4 tetrachloride with human telomeric RNA G-quadruplex was one order of magnitude stronger than that with DNA counterpart. This is a comprehensive and comparative mass spectrometric report on binding study of TMPyP4 with human telomeric DNA/RNA G-quadruplexes.

## Electronic supplementary material

Below is the link to the electronic supplementary material.

ESM 1 (PDF 209 kb)
