# Supplementary material for: Mass spectrometric studies on effects of counter ions of TMPyP4 on binding to human telomeric DNA and RNA G-quadruplexes
Source: Anal Bioanal Chem. 2014 Jun 18;406(22):5455–63. doi: 10.1007/s00216-014-7943-0 (PMC4141155; doi:10.1007/s00216-014-7943-0)
Supplement: Supplementary file 1 — (PDF 209 kb) [file 216_2014_7943_MOESM1_ESM.pdf]

Analytical and Bioanalytical Chemistry

Electronic supplementary material

Mass spectrometric studies on effects of counter ions of TMPyP4 on binding to human telomeric DNA and RNA G-quadruplexes

Li-Ping Bai , Jie Liu, Li Han, Hing-Man Ho, Renxiao Wang, Zhi-Hong Jiang

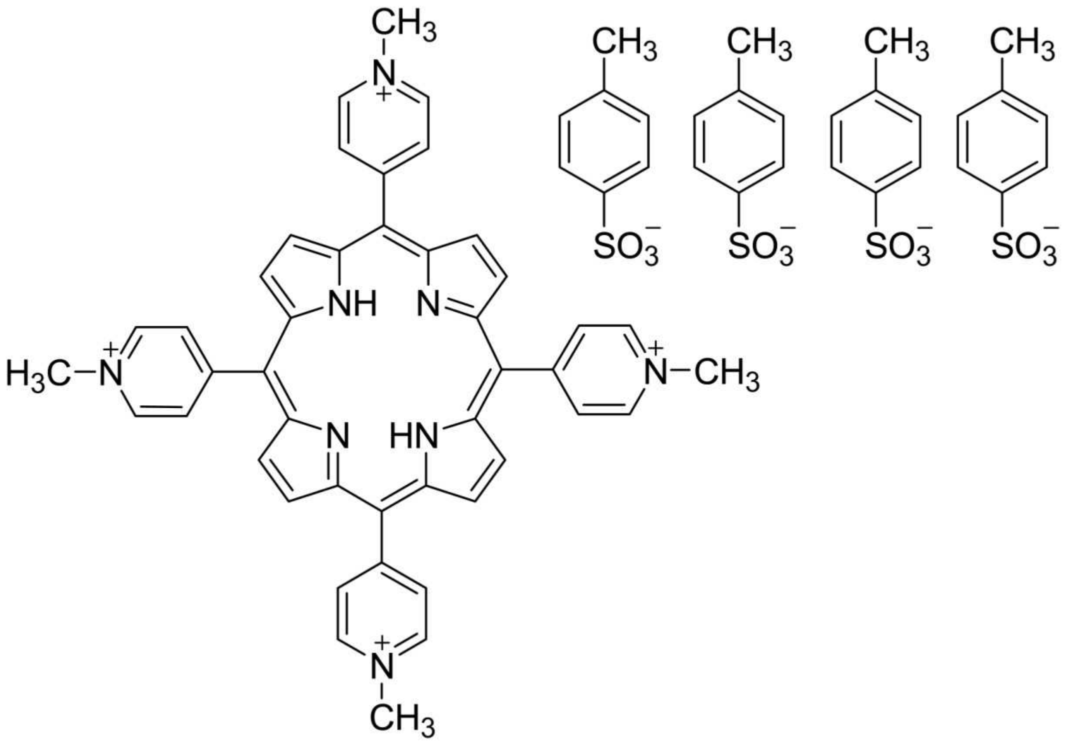

Fig. S1 Chemical structure of TMPyP4 tetratosylate

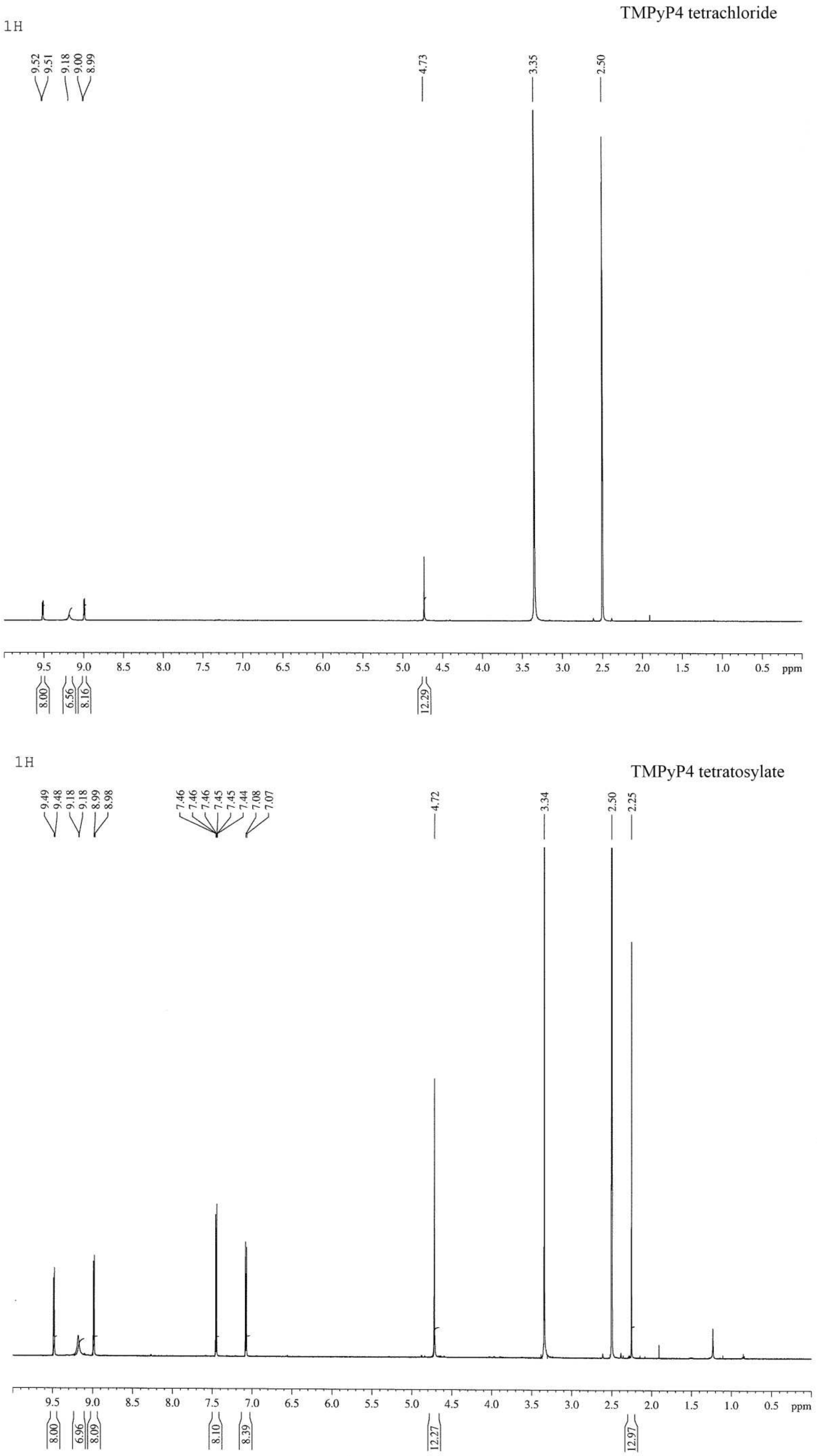

Fig. S2 ^{1}H-NMR spectra (600 MHz, DMSO-d_6) of TMPyP4 salts

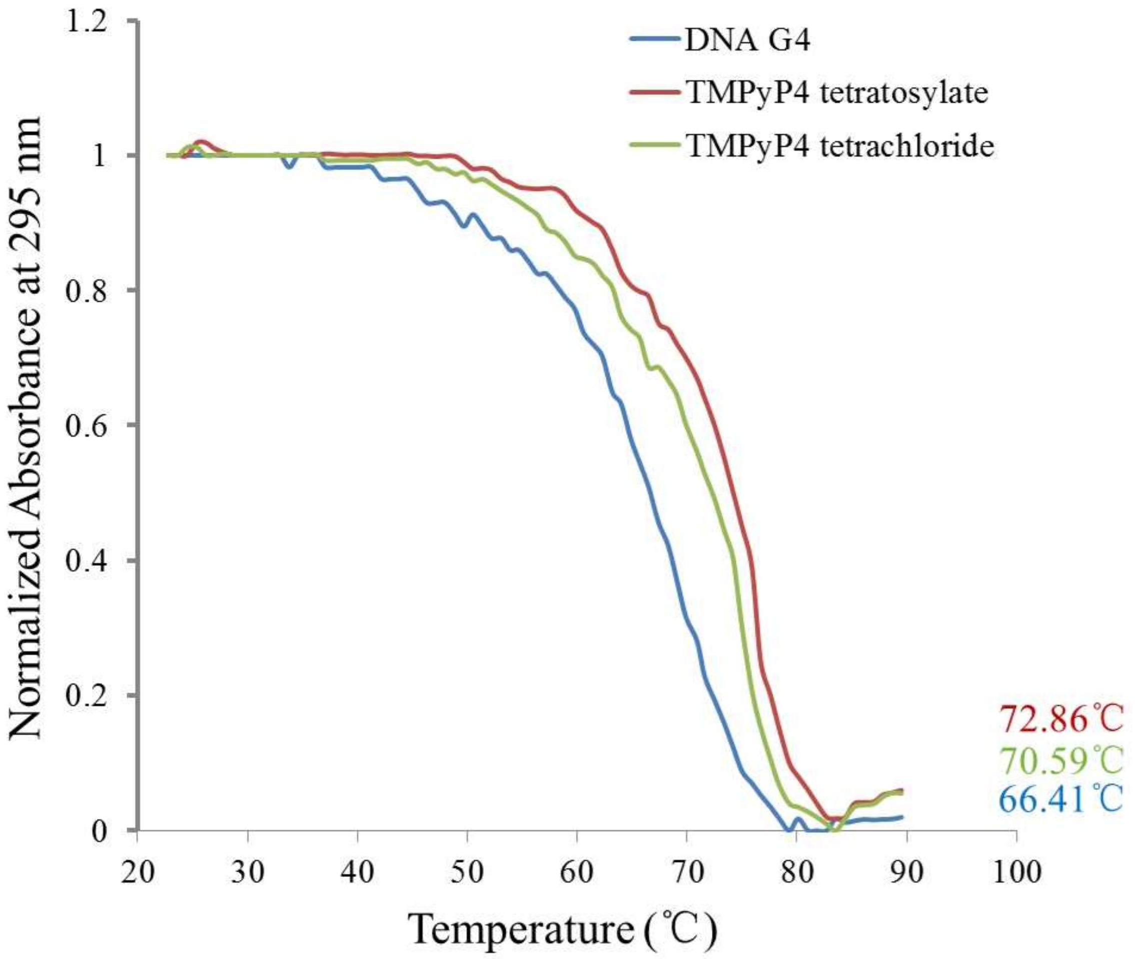

Fig. S3 Thermal denaturation profiles of DNA G-quadruplex (5 μM) in the absence and presence of TMPyP4 tetratosylate (10 μM) and TMPyP4 tetrachloride (10 μM) in 25 mM Tris-HCl buffer (pH 7.4) containing 100 mM KCl
